# Multicolor nanoring arrays with uniform and decoupled scattering for augmented reality displays

**DOI:** 10.1515/nanoph-2025-0410

**Published:** 2025-10-28

**Authors:** Inchul Woo, Hyeokjung Kang, Namkyoo Park

**Affiliations:** Seoul National University/LG Display Co., Ltd., Seoul, Korea; Seoul National University, Seoul, Korea

**Keywords:** transparent display, optical see-through, projection screen, spectrally selective, full-color, gamut

## Abstract

For vivid, immersive overlay of virtual images onto background scenes in augmented reality (AR) applications, it is crucial for the display element to achieve controllability of spectral selectivity and transmittance level. At the current stage, the transmittance of self-emissive transparent displays is limited to at most ∼60 %, constrained by the fill factor of emissive regions, restricting their scalability for immersive experiences. Although projection-based transparent screens using frequency-selective scatterers offer a promising alternative, the platforms suffer from spectral broadening and instability originating from color-dependent scattering and inter-scatterer coupling. Here, we present a transparent screen architecture based on multicolor nanoring arrays. By tuning the nanoring’s resonance via inner-aperture size engineering, the architecture enables dense, symmetric RGB arrays with isolated and homogenized scattering responses. For inter-scatterer distances of 100–200+ nm, full-wave simulations confirm the robustness of well-isolated RGB reflections (FWHM < 25 nm), along with exceptional tunability of transmittance (50 % to above 80 %). As a platform for AR displays, we demonstrate the widest reported transparency-control range without any penalty to color balance or spectral selectivity. We also analyze the gamut area of projected images across transmittance levels, achieving a net gamut expansion (+11.0 % p at Λ = 120 nm; +5.5 % p at Λ = 190 nm) from the spectral narrowing of projection sources, and further propose a practical design map linking the maximum allowable transmittance to the ambient-to-source noise ratio. Our nanoring-based architecture provides a robust and scalable platform for next-generation transparent displays under real-world lighting conditions.

## 1 Introduction

The increasing demand for immersive visual experiences – spanning augmented and virtual reality (AR/VR), smart infrastructure, and the broader metaverse ecosystem – has driven significant interest in transparent display technologies that seamlessly integrate digital content into the physical environment [1]. In AR applications, displays are expected to satisfy stringent requirements, including minimal bulk, low optical intrusion, and high-fidelity color rendering – criteria that pose challenges to conventional emissive and transmissive approaches [2]. Within this context, two major approaches have emerged for transparent displays. Self-emissive transparent displays offer compact pixel-level integration but require the coexistence of emissive and transmissive elements within the same pixel area. This spatial constraint typically limits transparency to ∼60 % in experimental implementations and creates scalability issues for high-resolution or large-area formats [3], [4], [5]. In contrast, projection-based transparent screens (TSs) offer flexibility in structural design and compatibility with external projection sources. However, they face an inherent trade-off between optical openness and image visibility: increasing transparency reduces scattering efficiency, thereby lowering color saturation and brightness.

To address these challenges, spectrally selective TS architectures have been proposed [6], [7], [8], [9]. These systems employ nanostructured scattering layers that reflect narrowband RGB wavelengths while transmitting the remainder of the visible spectrum, enhancing image clarity and preserving background visibility by minimizing spectral overlap. However, many prior designs rely on complex multilayer stacks, heterogeneous materials, or geometrically dissimilar scatterers – factors that complicate fabrication, introduce spectral imbalance, and limit uniformity over large areas. Although not demonstrated yet for transparent screens, all-dielectric color metasurfaces with their low loss could be of future interest. However, their narrow viewing angles, polarization sensitivities, and limited transparency – inherent to their high aspect-ratio, asymmetric structure [10] and high fill factors need to be mitigated [10], [11], [12]. Among various implementations, plasmonic nanodisks have shown promise due to their tunable resonances across the visible spectrum [13]. Yet achieving RGB selectivity in such systems typically requires using scatterers of dissimilar sizes, leading to two key drawbacks. First, at the single-particle level, differences in physical cross-sections lead to uneven scattering strengths and linewidths, often compromising spectral separation. Second, at the array level, size variation exacerbates near-field couplings and geometry-sensitive phenomena such as Fano interference [14], [15], resulting in reduced spectral fidelity, demanding frequent re-optimization, and exacerbating fabrication scalability.

**Figure 1:**
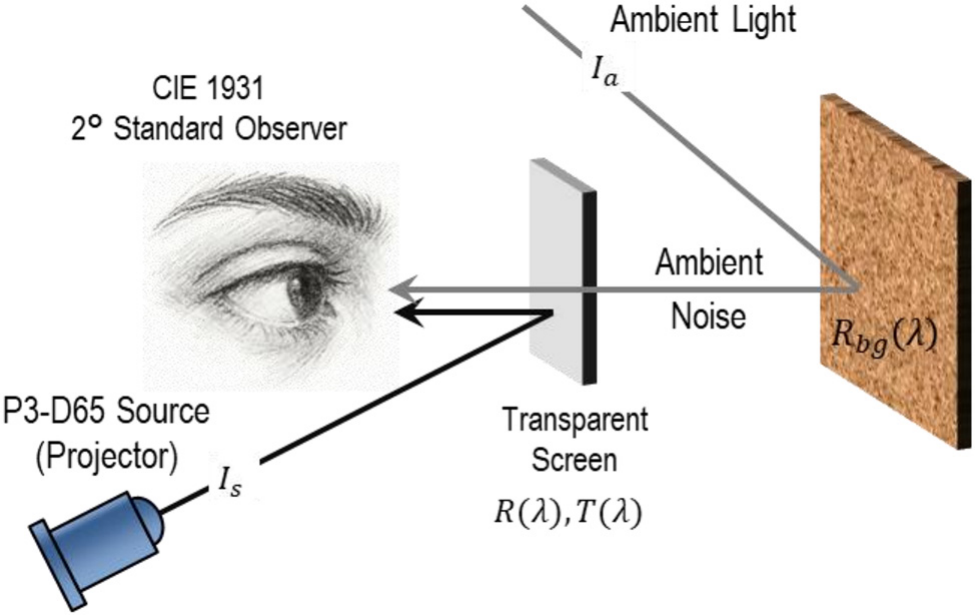
Schematic of the transparent AR/VR screen for a CIE 1931 2° standard observer. A P3-D65 projector provides the image signal *I*_*s*_ while ambient illumination *I*_*a*_ introduces background noise.

Here we propose a nanoring-based scattering architecture that enables RGB-selective reflection via modulation of the inner aperture size, yet at a fixed geometrical footprint across all color channels. The nanoring design leverages strong field confinement near the inner edge to maintain high spectral purity even in closely packed configurations, thereby preserving uniform scattering strength at the single-particle level and effectively suppresses inter-particle couplings, down to an inter-particle distance of 120 nm. In a transparent-screen configuration with a P3-D65 projection source and ambient illumination, the scatterers achieve gamut expansion relative to the source through spectral narrowing of the projection light. By extending nanoring applications – previously explored in sensing [16], [17], [18], [19], [20], [21], [22] and filtering [23], [24] – to transparent displays for the first time, this work delivers a fabrication-friendly, spectrally robust platform with tunable transparency, tolerance to layout variations, and high color fidelity for next-generation display technologies. A conceptual schematic of this AR transparent-screen configuration is shown in Figure 1.

## 2 Design principles and component behavior

### 2.1 Plasmonic scatterer design

**Figure 2:**
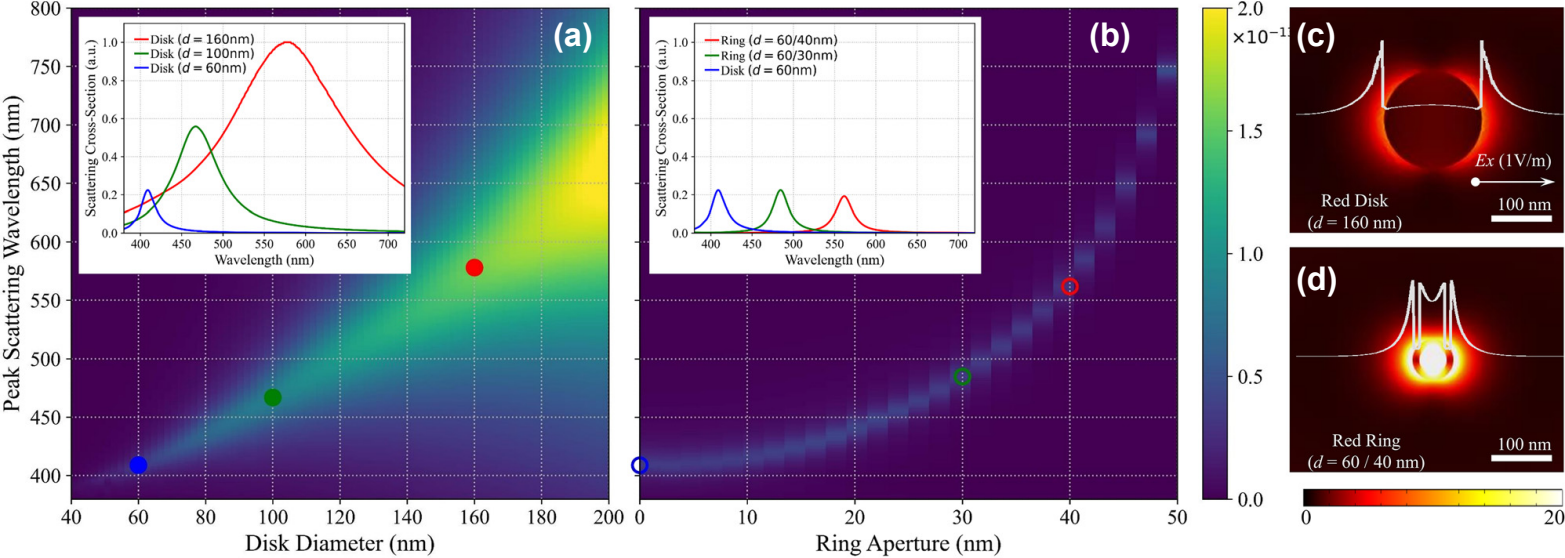
Geometry-controlled spectral tuning. (a) Simulated peak scattering wavelengths for nanodisks with varying diameters (*x*-axis), showing a redshift with increasing size. Inset: Scattering cross-sections for RGB-targeted disks (60, 100, 160 nm), indicating broad linewidths and spectral overlap. (b) Simulated peak scattering wavelengths for nanorings with fixed outer diameter (60 nm) and varying inner aperture sizes, demonstrating tunability by inner void size. Inset: Scattering cross-sections of RG-targeted nanorings (60/30, 60/40 nm) and 60 nm disk for blue, showing sharper, more spectrally separated peaks. (c–d) Electric-field magnitude (|*E*|) maps with cross-sectional profiles at resonance for the red-resonant nanodisk (160 nm) and nanoring (60/40 nm) under *x*-polarized excitation, illustrating distinct near-field confinement.

In conventional nano-plasmonic systems targeting practical applications, resonance wavelengths of the scatterers are usually controlled by a single parameter: the footprint of the particle. For example, nanodisks of distinct diameters can provide color-selective RGB resonances [10]. Despite its straightforward procedure for spectral design, this approach inherently leads to differences in physical footprint among color channels, resulting in variations in scattering cross-sections and resonance linewidths at the single-particle level. Figure 2 illustrates the comparative optical behavior of nanodisks and nanorings based on full-wave finite element method simulations. As shown in Figure 2(a), nanodisks exhibit a progressive redshift in peak scattering wavelength as the diameter increases, along with broader linewidths and larger scattering cross-sections – especially pronounced in the red channel, where the full width at half maximum (FWHM) exceeds 160 nm. The inset highlights the imbalance and spectral overlap of RGB-targeted disks (60, 100, and 160 nm), demonstrating the challenge of achieving consistent performance across color channels using footprint scaling.

To resolve the issue of wavelength engineering without altering the scattering cross-section, we employ a nanoring-based architecture in which a central aperture is introduced within the nanodisk geometry, offering an additional degree of freedom for spectral tuning. By varying the inner diameter while keeping the outer footprint constant, nanorings achieve tunable resonances across the visible spectrum – from blue to red – without altering the overall physical size. This size-invariant design ensures consistent structural parameters across RGB channels, yielding nearly uniform scattering cross-sections and improved spectral fidelity. As presented in Figure 2(b), nanorings with fixed *d*_out_ = 60 nm, *t* = 30 nm, and varying *d*_in_ = 0–50 nm, exhibit sharply defined, narrowband resonances at 409 nm (blue), 485 nm (green), and 562 nm (red), with FWHMs below 25 nm. The inset shows the scattering cross-sections of these RGB-targeted rings, contrasting with the broader spectral features of disks. In contrast to nanodisks, which exhibit redshifting and significant spectral broadening with increasing diameter, nanorings achieve consistent, narrowband RGB separation.

The improved spectral confinement observed in nanorings arises from hybridized plasmonic modes supported by their dual-edge geometry [25], [26], [27]. As the inner aperture expands, the effective optical path length increases – similar to scaling in optical cavities – resulting in a redshift of the resonance [25], [28]. Simultaneously, enhanced edge confinement maintains the radiative quality factor (Q-factor) through the suppressed higher-order multipolar excitations, thus resulting in narrower and more stable resonance peaks [26], [29], [30]. This distinction is visually evident in Figure 2(c) and (d), where the electric field profiles under resonant excitation show strong edge-localized modes in the nanoring, in contrast to the broader, dipolar distribution in the nanodisk. Together, these results demonstrate the superior performance of nanorings for compact, high-fidelity, and spectrally selective color filtering. The structural and spectral design parameters are further summarized in Table 1.

**Table 1:** Scattering of Ag nanodisks and – rings in air.

		Blue	Green	Red
Ag nanodisk	Diameter (nm)	60	100	160
	Peak wavelength (nm)	409	467	578
	Peak scattering cross-section (µm^2^)	3.5 × 10^−2^	8.7 × 10^−2^	1.6 × 10^−1^
	FWHM (nm)	20	63	163
Ag nanoring	Diameter (out/in, nm)	60	60/30	60/40
	Peak wavelength (nm)	409	485	562
	Peak scattering cross-section (µm^2^)	3.5 × 10^−2^	3.5 × 10^−2^	3.0 × 10^−2^
	FWHM (nm)	20	22	24

It is worth analyzing the robustness of the spectrum in view of fabrication tolerances. First, we note that a systematic peak bias can be remedied through a simple redesign to recenter the resonance. For random fabrication fluctuations, the red- and blue-shifted resonances result in an averaged spectrum for the film. Assuming fabrication errors up to *σ* ∼ 3 nm (10 % of the 30 nm aperture), the ensemble-averaged peak remains at the set point, with $\left|\Delta \lambda_{\text{peak}}\right|\leq 2\;\text{nm}$ , accompanied by spectral broadening and a reduction in peak scattering efficiency (∼50 %). Similarly, imperfections in fabrication could also introduce spectral changes, yet typically in the form of a negligible drift of the peak wavelength, a mild increase in loss, and a moderate FWHM broadening. Based on the above calculations and a separate analysis with an Ag defect (a 10-nm-diameter sphere), the averaged spectral peak shifts remain well below our target design resonance linewidth, especially given the large number of nanorings in a pixel (>500 in a 4-µm pixel in a conventional AR display) – in line with previous studies on the impact of imperfections in nanoring geometry [25], [26], [31], [32].

### 2.2 Inter-particle coupling suppression and array robustness

While spectral selectivity can be engineered at the single-scatterer level, practical implementation demands stability across diverse array configurations of constituting scatterers. In RGB arrays composed of size-mismatched scatterers, near-field interactions vary by channel, producing spacing-dependent resonance distortions and reduced spectral uniformity. These coupling effects complicate array design, limit scalability, and heighten sensitivity to fabrication tolerances – underscoring the need for architectures inherently robust in dense, multi-channel arrangements.

**Figure 3:**
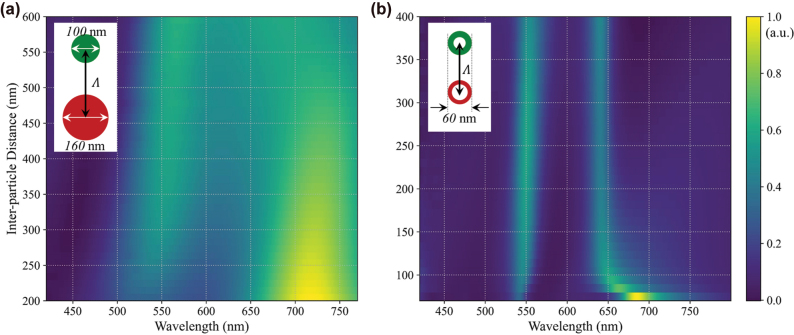
Inter-particle coupling effects in disk and ring dimers versus center-to-center spacing. (a) Simulated scattering spectra of disk dimers (160 nm red, 100 nm green) show strong spectral shifts and Fano-type interference at small separations. (b) Scattering spectra of ring dimers (60/40 nm red, 60/30 nm green) remain stable across spacings, indicating suppressed near-field coupling and greater layout tolerance. Color maps are normalized to their respective peak scattering intensities.

To examine this, we simulated dimer systems comprising either nanodisks or nanorings, with variable center-to-center spacing. As shown in Figure 3(a), disk dimers – particularly those representing the red and green channels – exhibit pronounced resonance shifts and linewidth broadening at reduced separations. These changes arise from strong dipolar coupling and Fano-type interference between size-mismatched elements, resulting in geometry-dependent spectral variations. In contrast, Figure 3(b) demonstrates that nanoring dimers maintain spectrally isolated, stable responses over a broad spacing range. Despite having distinct resonances, the scattering spectra remain largely invariant in peak position and linewidth, even at short inter-particle distances. This stability is attributed to strong modal confinement near the inner aperture, which limits the spatial reach of near-field interactions and thereby suppresses inter-particle coupling.

**Figure 4:**
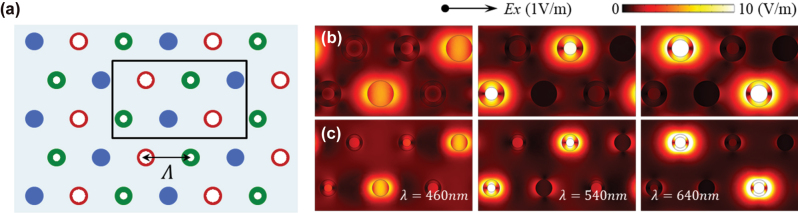
Array-level coupling suppression and field localization. (a) Schematic of an RGB-integrated array using uniform nanorings with tunable pitch, Λ. (b, c) Electric-field magnitude (|*E*|) maps at *λ* = 460 nm (blue), 540 nm (green), and 640 nm (red) for arrays with Λ = 120 nm and Λ = 190 nm. Fields remain channel-specific and spatially localized, confirming spectral separation is preserved across layout densities.

Extending this analysis to full RGB arrays in a hexagonal layout (Figure 4(a)), we evaluate near-field distributions for array pitches (Λ) of 120 nm and 190 nm under *x*-polarized illumination at representative RGB wavelengths. As shown in Figure 4(b) and (c), electric fields remain tightly localized around each nanoring, with minimal overlap between neighboring elements. This confirms that spectral separation and channel fidelity are preserved even in densely packed configurations. By maintaining spectral performance independently of array density, nanoring-based scatterers eliminate the need for additional buffer space or asymmetric layouts for decoupling, significantly simplifying the design of the multicolor array film. Such layout-independent spectral stability is essential for scalable, large-area TSs, enabling high-density, geometry-invariant integration without performance degradation.

## 3 Optical performance evaluation

### 3.1 Spectral stability under transparency modulation via array pitch

Building on this, we now examine whether these advantages persist under global pitch modulation, where inter-particle spacing is varied to control the optical transparency of the screen – a critical factor, as pitch variation is one of the most fabrication-accessible parameters for real-world TS tuning, directly influencing broadband reflectance, RGB spectral profiles, and color balance. Increasing the center-to-center spacing between scatterers reduces the areal fill factor, thereby increasing transmittance but correspondingly lowering reflectance and absorbance.

**Figure 5:**
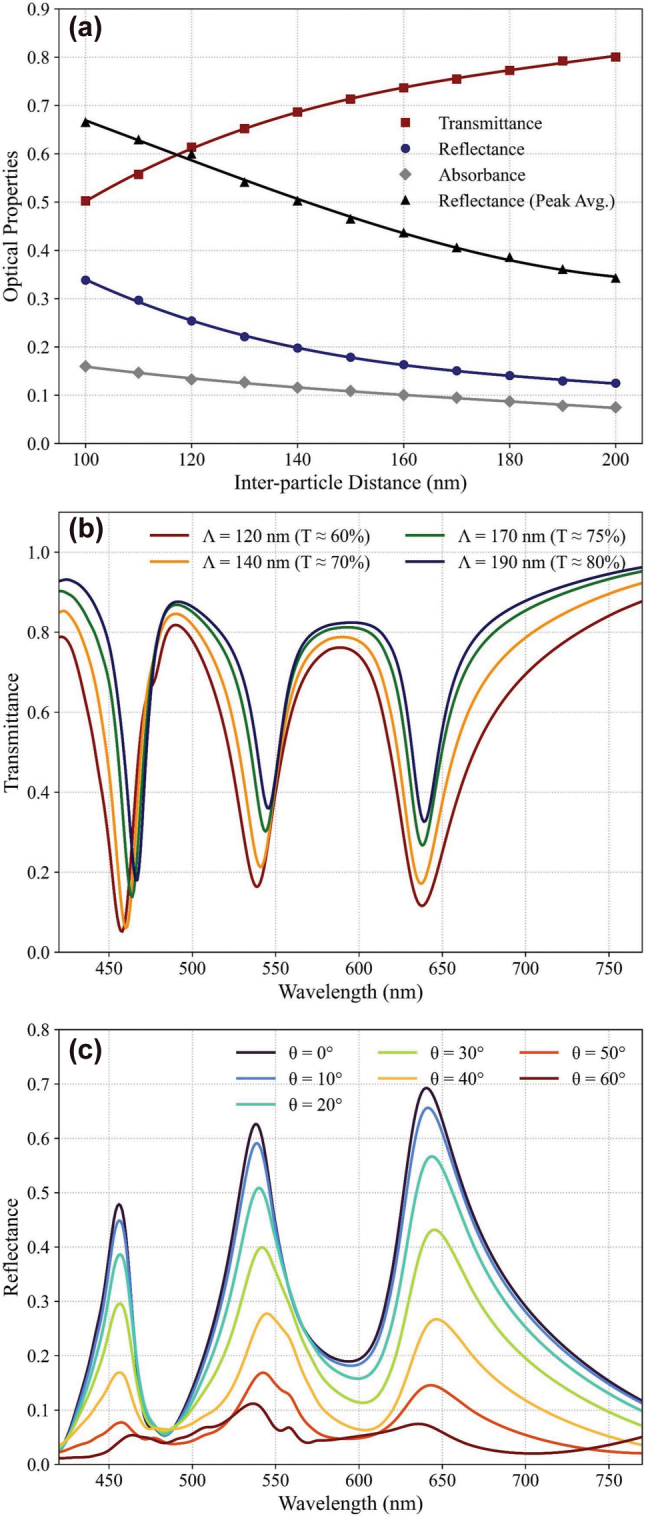
Optical response under pitch-modulated transparency and viewing-angle robustness. (a) Simulated broadband transmittance (*T*), reflectance (*R*), and absorbance (*A*) as functions of array pitch. The “Peak Avg.” metric represents the mean RGB peak reflectance, highlighting spectral selectivity. (b) Full transmission spectra for Λ = 120, 140, 170, and 190 nm (*T* ≈ 60–80 %), demonstrating consistent RGB peaks and color balance across transparency levels. (c) Specular-reflectance spectra of the RGB nanoring array (Λ = 120 nm) for incidence angles *θ*_inc_ = 0–60°.

Figure 5(a) summarizes these results by plotting transmittance (*T*), reflectance (*R*), and absorbance (*A*), averaged over the visible spectrum (380–780 nm), as functions of array pitch. These results indicate that varying the pitch allows continuous adjustment of the screen’s optical properties while keeping the scatterer geometry fixed, providing a straightforward means of transparency control. Here, “Peak Avg.” denotes the arithmetic mean of the peak reflectance values measured at the RGB target wavelengths, defined as $R_{\text{peak,Avg}}=\left(R_{R}^{pk}+R_{G}^{pk}+R_{B}^{pk}\right)/3$ . This metric intuitively captures the strength and spectral sharpness of each color channel, independent of broadband reflection. A higher peak avg. indicates enhanced color saturation and serves as a reliable predictor of extended color gamut. Its consistently high values – more than twice the average broadband reflectance across all tested configurations – confirm that vivid, saturated color reproduction is maintained even at higher transparency levels.

Figure 5(b) presents full transmission spectra for four representative pitches: Λ = 120, 140, 170, and 190 nm (corresponding to *T* ≈ 60–80 %). Across this range, distinct reflection peaks remain well-defined and spectrally isolated, with minimal shifts in peak position or linewidth. The relative amplitudes across RGB channels also remain well-balanced, confirming that spectral fidelity and inter-channel balance are preserved across the transparency tuning range.

Figure 5(c) shows specular reflectance spectra of the RGB nanoring array (Λ = 120 nm) for incident angles *θ*_inc_ = 0–60°. The RGB resonance wavelengths exhibit negligible drift (|Δ*λ*| ≤ 5 nm) up to |*θ*_inc_| ∼ 50°, and the peak amplitudes decrease smoothly with increasing angle of incidence (reaching half their values at *θ*_inc_ ∼ 35°). This robustness to viewing angle is attributed to the nanorings’ symmetry, low aspect ratio, and deep-subwavelength footprint. With an acceptance >70°, we note that our film meets the field-of-view (FoV) requirements of typical optical see-through AR displays.

These results verify that nanoring arrays enable continuous modulation of optical transparency via pitch adjustment with minimal peak shifts or linewidth changes. The ability to decouple transparency control from spectral fidelity offers greater flexibility for see-through display applications, allowing dynamic optimization of background visibility and image quality according to specific usage scenarios.

### 3.2 Gamut expansion and design applications under ambient light

Beyond tunable transparency, a critical – yet often overlooked – performance metric for TSs is their ability to maintain a wide and stable color gamut under ambient illumination. Test conditions assume a worst-case outdoor scenario (∼10,000 lux) modeled by the CIE D65 [33], [34]. Under this illumination, a 2,000 ANSI lumens projection source tightly focused onto a small AR screen area yields a noise ratio (ambient/source) of approximately 0.5 %, whereas reduced focusing efficiency proportionally increases this ratio, further challenging color fidelity [35]. In conventional spectrally flat transparent screens, ambient light mixes linearly with the RGB primaries and pulls chromaticity toward the white point in the CIE diagram, thereby reducing saturation and compressing the effective gamut under the standard color-metrology/IDMS framework [33], [36]. This effect is particularly severe in see-through displays, where ambient leakage into the optical path is unavoidable.

To isolate the impact of spectral selectivity of the suggested transparent screen, we modeled an RGB source with Gaussian primaries as listed in Table 2, matched to the P3-D65 standard with chromaticities within ±0.005, and used the resulting gamut as the reference for all comparisons [37]. For the signal and ambient noise following the paths in Figure 1, *I*_sig_ = *R*(*λ*)*I*_*s*_(*λ*) and $N\left(\lambda \right)=I_{a}\left(\lambda \right)R_{bg}T\left(\lambda \right)$ , where $I_{a}\left(\lambda \right)$ is the normalized D65 spectral power distribution and *R*_bg_ = 0.18 is set to the fixed reflectance of the background scene (assumed standard middle gray [38]). The perceived spectrum with ambient noise can be written as $I_{\text{tot}}=I_{\text{sig}}\left(\lambda \right)+N\left(\lambda \right)$ , which is integrated with the CIE 1931 2° color-matching functions at matched transmittance. The proposed nanoring screen successfully and effectively mitigates ambient contamination via multiplicative spectral sharpening, when its reflection peaks are closely aligned with the source primaries. It is emphasized that the narrowing of the RGB source spectra from the convolution of RGB nanoring increases chromatic separation in the CIE plane, and can even yield a net gamut expansion relative to the source in the low-noise limit (e.g., in the regime of ambient/source < 0.2 % and 1.2 %, for *T* = 80 % and 60 %). This mechanism and its parameterization are consistent with the pitch-modulated behavior established previously.

**Table 2:** Parameters for the simulated RGB light source modeled with Gaussian primaries: $I\left(\lambda \right)=I_{o}\cdot \exp\left[-{\left(\lambda -\lambda_{o}\right)}^{2}/\left(2\sigma^{2}\right)\right]$ , matched to P3-D65 within ±0.005 in chromaticities, with the resulting gamut used as the comparison reference.

		*R*	*G*	*B*	*W*	Gamut area
Simul. source	*I*_o_	0.86	0.76	0.63	–	0.152
	*λ*_o_	645	538	450	–	
	*σ*	27.4	22.3	30.5	–	
	*x*	0.679	0.262	0.144	0.313	
	*y*	0.321	0.686	0.059	0.328	
Ideal P3-D65	*x*	0.680	0.265	0.150	0.313	0.152
	*y*	0.320	0.690	0.060	0.329	

**Figure 6:**
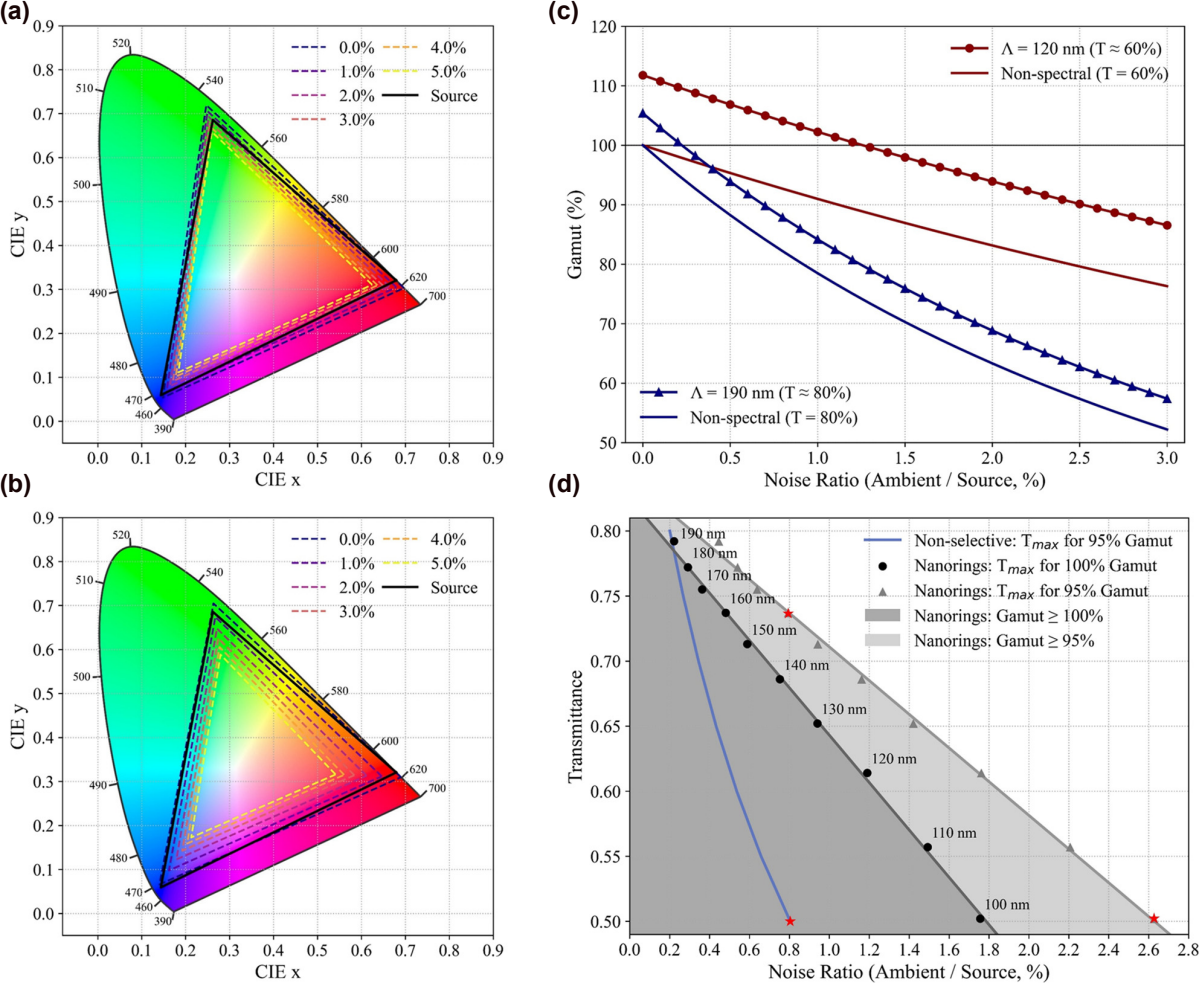
Gamut expansion and design applications under ambient light. CIE *xy* chromaticity diagrams for nanoring arrays with (a) Λ = 120 nm and (b) Λ = 190 nm under increasing ambient D65 illumination. The black triangle indicates the source gamut and the colored triangles denote the resulting gamut. (c) Normalized gamut area relative to the source showing an enhanced offset from a non-spectral baseline at the same transmittance due to reflection sharpening, with +11.0 % p at 120 nm and +5.5 % p at 190 nm. (d) Design map of the maximum allowable transmittance versus noise ratio, with the corresponding pitch indicated, for two gamut-retention targets (100 % and 95 %). Since the non-selective screen cannot satisfy 100 % of the source gamut for any non-zero noise ratio, its curve collapses at the *y*-axis and is omitted.

Figure 6(a) and (b) show CIE *xy* diagrams for nanoring arrays with pitches of 120 nm and 190 nm under increasing D65 ambient illumination. The black triangle indicates the source-only gamut, while the colored triangles represent the resulting gamuts with ambient light included. At low to moderate ambient levels, both configurations exceed the source gamut, consistent with sharpening arising from the narrow resonance linewidth enabled by the nanoring design. Figure 6(c) quantifies this behavior by plotting the normalized gamut relative to the noise ratio. Across the evaluated range, the curves exhibit an upward offset with respect to a non-selective baseline at matched transmittance, attributable to reflection sharpening: +11.0 % p for Λ = 120 nm and +5.5 % p for Λ = 190 nm. This framing emphasizes that the benefit manifests as a vertical expansion (offset) relative to a non-selective counterpart.

Further leveraging the spectral filtering and corresponding gamut-expansion capability of the proposed transparent screen, we also optimize the screen’s transmittance to deliver the best projection quality across application scenarios spanning a range of noise ratios. As shown in Figure 6(d), taking 95 % of the source gamut as the target image quality, we can maintain the projected image quality by adjusting the film transmittance together with the corresponding pitch, up to a noise ratio of 2.6 % in the extreme. It is emphasized that this value is ∼330 % higher than the 0.8 % noise tolerance of a conventional flat-spectral film, and it also implies that at the same noise ratio of 0.8 %, our film can provide higher transparency (∼74 %) than the flat-spectral film (∼50 %).

## 4 Conclusions

We have proposed a nanoring-based plasmonic scattering architecture that addresses key challenges in TS design, including spectral instability, geometric asymmetry, and scalability limits of conventional nanostructures. By achieving RGB-selective scattering through aperture-tuned, size-invariant nanorings, this structure ensures balanced color performance while enabling uniform, layout-flexible integration.

The system supports transparency tuning via global pitch modulation across Λ = 100–200+ nm, maintaining narrowband RGB reflections and stable channel separation even as the fill factor changes. Across this range, color balance and spectral selectivity remain consistent, with FWHM < 25 nm and peak-averaged reflectance exceeding twice the broadband reflectance, yielding transmittance from 50 % to well above 80 % without spectral penalty. Under ambient illumination, spectral sharpening mitigates gamut loss and, at low to moderate ambient levels, the resulting gamut surpasses the source gamut area. Leveraging this gamut-expansion behavior, we delineate operating regimes that balance ambient illumination, transmittance, and projector primaries to maintain source coverage and enable >100 % extension when feasible; building on this, we provide a practical design map that links the maximum allowable transmittance to the noise ratio.

For large-scale experimental realization of the film, two complementary routes can be considered. For self-assembly, colloidal lithography has been used to fabricate wafer-scale arrays of single-size Au nanorings [22] and Ag coaxial cavities [39]. As a straightforward extension, area-selective hole-mask colloidal lithography [40] can be applied to realize nanorings of different sizes and colors. Alternatively, leveraging e-beam lithography demonstrated for multi-size Au and Si nanorings [17], [41], one could replicate the EBL mask at wafer scale using UV-NIL, providing narrow-gap capability [42] and wafer-scale templating [43].

By combining tunability and robustness under layout/ambient variations, the proposed architecture offers a fabrication-friendly, scalable platform for next-generation transparent displays suited for AR, HUDs, and immersive visual systems where both optical clarity and high-fidelity color reproduction must be maintained in real-world lighting conditions.
